# Few incidentally found interesting foreign objects in human body: a case series

**DOI:** 10.12688/f1000research.134773.2

**Published:** 2024-06-06

**Authors:** ANAND HATGAONKAR, KAJAL HATGAONKAR, SANDEEP DHOTE, VAISHALI DHAWAN

**Affiliations:** 1 Radiodiagnosis, Datta Meghe Medical College, Nagpur. Datta Meghe Institute of Higher Education and Research (DU), Swangi (Meghe), Wardha, Maharashtra, 442107, India; 2 Pathology, Datta Meghe Medical College, Nagpur. Datta Meghe Institute of Higher Education and Research (DU), Sawangi (Meghe), Wardha, Maharashtra, 442107, India; 3 Radiodiagnosis, NKP Salve Institute of Medical College and Research, Nagpur, Maharashtra, 440016, India; 4 Radiodiagnosis, Jawaharlal Nehru Medical College, Datta Meghe Institute of Higher Education and Research (DU), Sawangi (Meghe), Wardha, Maharashtra, 442107, India

**Keywords:** foreign bodies, ingestions, insertions, injuries, X-ray, CT scan, ultrasound, MRI.

## Abstract

Foreign bodies are objects that do not typically belong in the human body but can be ingested, inserted, or entered due to injuries. This article presents various cases and examples of foreign bodies, including objects swallowed, objects inserted into the rectum, vagina, urethra, ear, and nose, or due to injuries caused by falls, puncture wounds, and gunshot wounds.

Foreign bodies can be difficult to detect, particularly if they are not inherently radio-opaque, and may be overlooked by patients who cannot provide an adequate history. These foreign bodies may cause harm to the patient. Interpretation is done on radiographs, computed tomography (CT), Ultrasonography (USG), and magnetic resonance imaging (MRI) studies.

Most foreign objects pass through the gastrointestinal tract without problem; sharp and elongated objects can cause significant injury, and even if they only partially perforate the bowel wall, they can produce chronic inflammatory processes that produce symptoms months or years later. Hence, searching for foreign bodies should be done throughout the gastrointestinal tract, particularly in children and people with mental illness who are more likely to swallow multiple items more than once.

Although rare, various materials can be left behind in the body of a patient after surgery, including large and small wire sutures, surgical drains, and retained sponges, which can cause potential complications and foreign body reactions.

This article highlights the importance of being aware of the presence of foreign bodies in clinical practice, and a thorough search should be carried out using different modalities, especially CT. Great suspicion and early diagnosis of foreign bodies can avoid potential complications and morbidity. In general, it provides information on the diagnosis and treatment of various types of foreign bodies.

## Introduction

The human body is and forever will be an amazing mystery. But sometimes it is even more surprising to find things that do not normally belong in a human body, like a pen refill in the stomach or a sharp metallic object in the bladder. Although they are rare, foreign bodies are fascinating and significant. They can be overlooked and can cause harm to the patient. If one does not suspect the presence of a foreign body, interpreting radiograph, computed tomography (CT), ultrasonography (USG), and magnetic resonance imaging (MRI) investigations are especially prone to inaccuracy. ^1^ Children, mentally challenged people, adults who exhibit atypical sexual behavior and even “normal” adults or children with risk factors are more likely to consume or introduce foreign bodies. ^2^

This article discusses key concepts about foreign body ingestions, insertions, and injuries while illustrating a range of shocking foreign bodies.

**Methods:** This case series was carried out at tertiary care centre in central India. Radiography was done on digital and computerized radiography X-ray machine; multi-slice CT scanner and 1.5 Tesla MRI.

**Ethical consideration:** All ethical principles were followed during the study and all measures are taken to maintain anonymity. Institutional ethical committee of Shalinitai Meghe Hospital and research center, which is constituent unit of Datta Meghe Medical College have granted ethical clearance for study vide letter no. SMHRC/IEC/2023/02-59 dated 17/02/2023.

**Consent:** Written informed consent for publication of their clinical details and/or clinical images was obtained from the patient/parent/guardian/relative of the patient.

## Cases

**Case 1:** A four-year-old child came with a history of abdominal pain for 5–6 hours. A clinical examination revealed soft to touch abdomen that was unremarkable. On a radiograph of the abdomen, frontal and lateral projections reveal circular radio opacity on the left side at the level of the L2-L3 disc suggestive of coin, which the patient had accidentally ingested. It passed through the gastro-intestinal tract without a problem.

**Case 2:** A 34-year-old male carpenter by profession accidentally ingested a screw before 3 days. He had mild, intermittent pain in the right iliac fossa. The clinical examination revealed no significant findings. A radiograph of the abdomen in frontal and lateral views reveals a nail at the level of the L4 and L5 vertebrae in the gastrointestinal tract, which passed without any problems.

**Case 3:** A 25-year-old male patient came for ultrasound examination with complaints of pain in the abdomen for two-three months. On palpation of the abdomen, the patient had tenderness in the epigastric and periumbilical regions. The clinical examination of the rest of the systems was normal. Radiograph of the chest and abdomen reveal multiple linear radio-opacities in the left hypochondriac and lumbar quadrants of the abdomen, and a plain CT scan shows multiple hyperdense linear metallic foreign bodies within the gastric lumen, many piercing the gastric wall partially without any evidence of perforation. On laparoscopy, multiple refills of the pen and wires were found in the stomach, which were removed.

**Case 4:** An 18-year-old woman patient came with a history of abdominal pain and vomiting on and off for 15 days. On physical examination, the patient had tenderness in the epigastric region with a palpable lump. Visible patchy hair loss is noted on the scalp. Contrast enhanced computed tomography (CECT) reveals a heterogeneous lamellated non-enhancing soft tissue density mass (with a wide attenuation range from -70 to 70 HU) intraluminally in the stomach, conforming to its shape and extending into the antrum, pylorus, and minimally into the duodenal cap suggestive of trichobezoar. Gastrotomy revealed the ball of hair in the stomach.

**Case 5:** A seven-year-old boy came with a history of epistaxis for a day. On clinical examination, foul-smelling, blood-tinted nasal discharge was noted on the left side. Further examination was not possible as the child was uncooperative. CT paranasal sinuses (PNS) revealed a non-enhancing hyperdense lesion in the left nasal cavity, possibly a foreign body. It was removed under anaesthesia and found to be a castor seed.

**Case 6:** A 17-year-old woman came for an ultrasound examination in emergency hours with complaints of severe pain in her lower abdomen. On clinical examination, she had tenderness in the hypogastric region on palpation and no other significant contributing findings. Radiograph Pelvis anteroposterior (AP) view revealed long radio-opacity in the bladder with a radiolucent center that did not look like a calculus, but a foreign body. USG revealed a linear hyperechoic foreign body that penetrated the anterior wall of the bladder. The patient had a history that she had conceived three years ago and had tried abortion by a quack in her village. The patient was operated on and a shaggy piece of a long wooden stick with cotton wrapped around it was found.

**Case 7:** A 40-year-old male presented with a complaint of pain in the right heel region for two months. On examination, he had mild, hard swelling on the posterior part of the ankle. A radiograph of the lateral view of the right foot revealed there was evidence of calcific tendinitis of the Achilles tendon with thickening of the Kager fat pad and fat stranding. USG revealed that a well-defined thorn visualized in the Achilles tendon with associated surrounding tendinitis and increased fat echogenicity. USG-guided thorn removal was performed.

**Case 8:** A 50-year-old woman came for cervical spine. The patient complained of neck pain and headache for more than 1 year. The clinical examination was unremarkable. The patient was taken for an MRI scan when she complained of a severe headache. Radiograph skull AP & Lateral view was done, which showed a radiodense nail-like structure in scalp on right side. On asking, the patient gave a history of trauma ten years back and did not know that she had a nail in her scalp. It was removed under local anaesthesia.

**Case 9:** A 37-year-old man had a history of bullet injury three years back. On examination, multiple small penetrating wounds were seen in the region of the thorax and abdomen. Radiographs of the chest and abdomen in frontal and lateral views revealed multiple pellets in the subcutaneous soft tissue of the thorax and abdomen.

**Case 10:** A 33-year-old female came with a history of bleeding pervagina for six months. On palpation, the patient had tenderness with mild guarding in the hypogastric region. The rest of the clinical examination was non-contributory. MRI shows heterogeneous altered signal intensity soft tissue mass anterior to the uterus with multiple hypointense foci within. CT showed multiple linear metallic strings within a mass of soft tissue density anterior to the uterus, suggesting a foreign body (gossypiboma). The patient was operated on, and a large surgical sponge was removed.

## Result

A total of ten cases were studied, comprising of four females and six males of various age groups ranging from four years to 50 years ( Tables 1 & 2). Four patients had ingested foreign bodies while two patients had history of insertion and four other had insertion due to injury ( Table 3).

**Table 1.** Case wise distribution.

Case No.	Age/Sex	Way of entry of FB inside the body	Type of FB	Imaging modality used for diagnosis	Imaging findings	Intervention used for removal of FB
1.	4 yr/M	Accidental Ingestion	Coin	Radiograph of the abdomen, frontal and lateral projections	circular radio opacity on the left side at the level of the L2-L3 disc s/o coin.	No intervention needed, the coin was passed in stool.
2.	34yr/M	Accidental Ingestion	Nail	Radiograph of the abdomen, frontal and lateral projections	A nail at the level of the L4 and L5 vertebrae in the gastrointestinal tract.	No intervention needed, the nail was passed in stool.
3.	25yr/M	Ingestion	Pen-refills and wires	Radiographs of the chest and abdomen and	Multiple linear radio-opacities in the left hypochondriac and lumbar quadrants of the abdomen.	Laparoscopic removal of the multiple refills of the pen and wires from stomach.
				Plain CT scan of abdomen	Multiple hyperdense linear metallic foreign bodies within the gastric lumen, many piercing the gastric wall partially without any e/o perforation.	
4.	18yr/F	Ingestion	Hair	Contrast enhanced Computed tomography (CECT)	Heterogeneous lamellated nonenhancing soft tissue density mass (with a wide attenuation range from -70 to 70 HU) intraluminally in the stomach, conforming to its shape and extending into the antrum, pylorus, and minimally into the duodenal cap s/o trichobezoar.	Ball of hair in the stomach was removed during Gastrotomy.
5.	7yr/M	Insertion	Castor seed	CT scan of Paranasal sines	Non-enhancing hyperdense lesion in the left nasal cavity, possibly a foreign body.	The seed was removed under anaesthesia.
6.	17yr/F	Insertion	Long wooden stick with cotton wrapped around it	Radiograph Pelvis A-P view	Long radio-opacity in the bladder with a radiolucent center that did not look like a calculus, but a foreign body.	Operated under anaesthesia and the FB removed.
				USG	A linear hyperechoic foreign body that penetrated the anterior wall of the bladder.	
7.	40yr/M	Insertion due to injury	Thorn	Radiograph of the lateral view of the right foot	e/o calcific tendinitis of the Achilles tendon with thickening of the Kager fat pad and fat stranding.	USG-guided removal of thorn.
				USG	A well-defined thorn visualized in the Achilles tendon with associated surrounding tendinitis and increased fat echogenicity.	
8.	50yr/F	Accidental insertion during episode of trauma 10 years back	Nail	Radiograph skull A-P & Lateral view	A radiodense nail-like structure noted in scalp on right side.	Removed under local anaesthesia.
9.	37yr/M	Insertion during bullet injury	Multiple pellets	Radiograph of the chest and abdomen in frontal and lateral views	Multiple pellets in the subcutaneous soft tissue of the thorax and abdomen.	Removed under local anaesthesia.
10.	33yr/F	Accidental insertion during surgery	Large surgical sponge	CT scan of pelvis	Multiple linear metallic strings within a mass of soft tissue density anterior to the uterus, suggesting a foreign body (gossypibomas).	Operated under general anaesthesia.
				MRI pelvis	Heterogeneous altered signal intensity soft tissue mass anterior to the uterus with multiple hypointense foci within.	

FB - foreign body, Yr - years, M- male, F - female, CT - Computed tomography, CECT - Contrast enhanced computed tomography, USG - ultrasonography, e/o - evidence of, AP - anterior-posterior, MRI - magnetic resonance imaging.

**Table 2.** Demographic distribution.

Age distribution	Male	Female	Total No. of patients
<10 years	02	00	02
11-20 years	00	02	02
21 to 30 years	01	00	01
31 to 40 years	03	01	04
41 to 50 years	00	01	01
Total	06	04	10

**Table 3.** Distribution according to possible way of entry.

Way of entry of foreign body	No. of patients	Age range	Male	Female
**Ingestion**	4	4 to 34 yrs	3	1
**Insertion**	2	7 to 18 yrs	1	1
**During Injury**	4	33 to 50 yrs	2	2

## Discussion

Foreign bodies are objects that do not typically belong in the human body but can be ingested, inserted, or entered due to injuries. This article presents various cases and examples of foreign bodies, including objects swallowed, objects inserted into the rectum, vagina, urethra, ear, and nose, or due to injuries caused by falls, puncture wounds, and gunshot wounds.

Ingestion or insertion of foreign bodies is commonly seen in children of all ages and mentally challenged individuals. Accidental insertion of the foreign bodies due to injuries is commonly seen in musculoskeletal region. The patient's risk of aspirating a foreign body increases if they have neurological impairment, facial trauma, dental instrumentation, or are intubated. Drug packing is a well-researched type of foreign body insertion that involves swallowing or inserting medications anally or vaginally while they are covered in foil, latex, or cellophane.

Radiography is initial screening modality and modality of choice for radio-opaque foreign bodies. At least two perpendicular views are recommended for proper localization of radio-opaque foreign bodies. The advantage of a CT scan is its ability to identify small radio-opaque foreign bodies as well as their position and closeness to important structures, including the involvement of neurovascular bundles. It can more clearly show granulomas, related inflammation, and the development of abscesses—all of which are secondary indicators of a retained foreign substance. When radiography is negative, ultrasound is particularly helpful in the case of musculoskeletal radiolucent foreign bodies, which can be more accurately targeted and can be removed under ultrasound guidance. By identifying the granulation tissue surrounding foreign objects or the presence of metal or air in the form of a susceptibility artifact, MRI can be helpful. Although it is ineffective when it comes to metallic foreign bodies because of ferromagnetic streak artifacts and can exacerbate existing injuries due to displacement, magnetic resonance imaging (MRI) can be useful in certain situations when evaluating granulomatous alterations, neurovascular involvement, and infection associated with foreign bodies. If vascular involvement is suspected, CT or MR angiography may be advised. A comprehensive physical examination, a high degree of clinical suspicion, and the appropriate choice of the suitable imaging modalities will yield the best results, as no single modality is perfect for identifying foreign bodies.

### Foreign body ingestions

**Figure 1.**
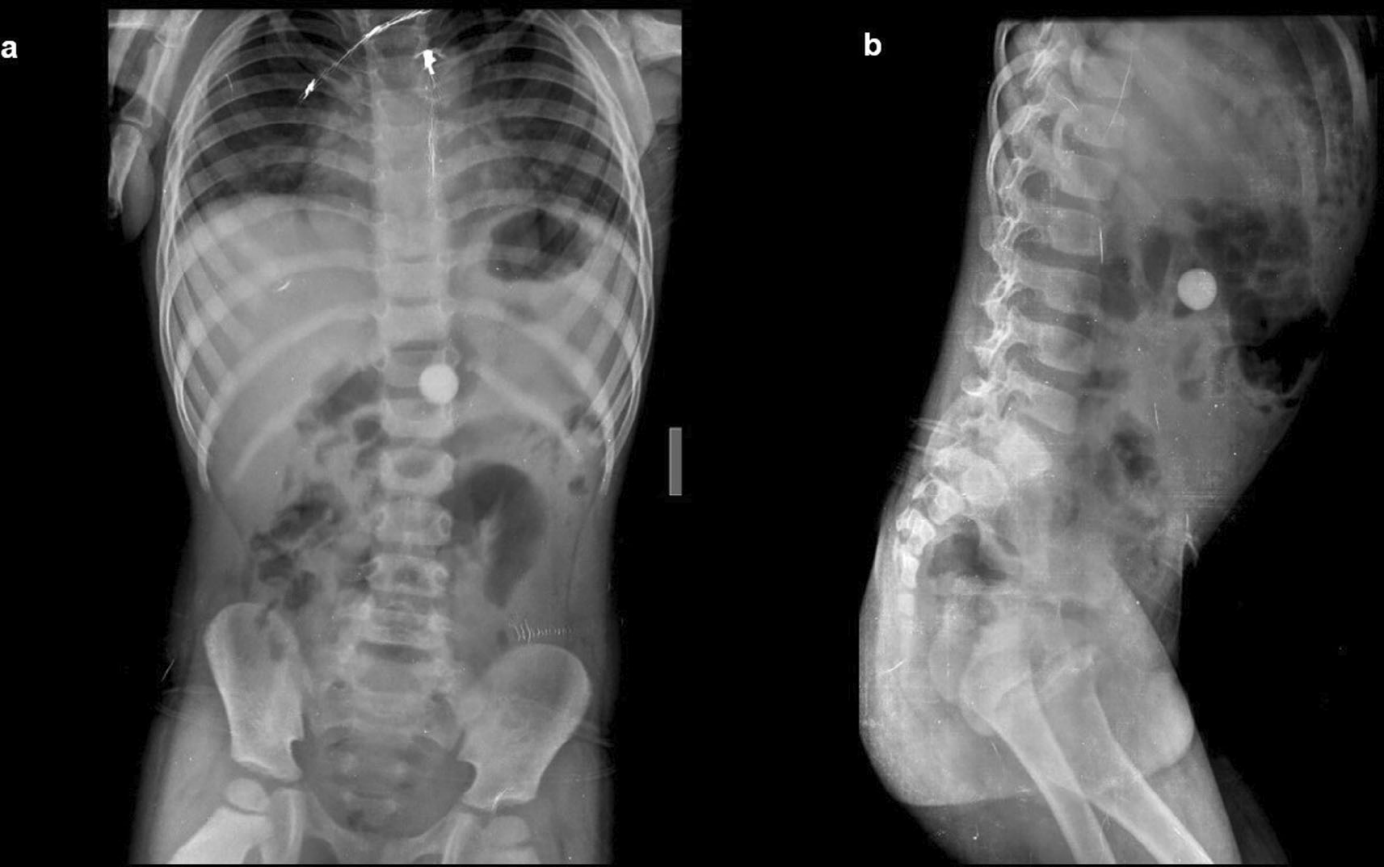
a & b: Radiograph of the abdomen, frontal and lateral projections reveal circular radio opacity on the left side at the level of the L2-L3 disc s/o foreign body likely coin.

**Figure 2.**
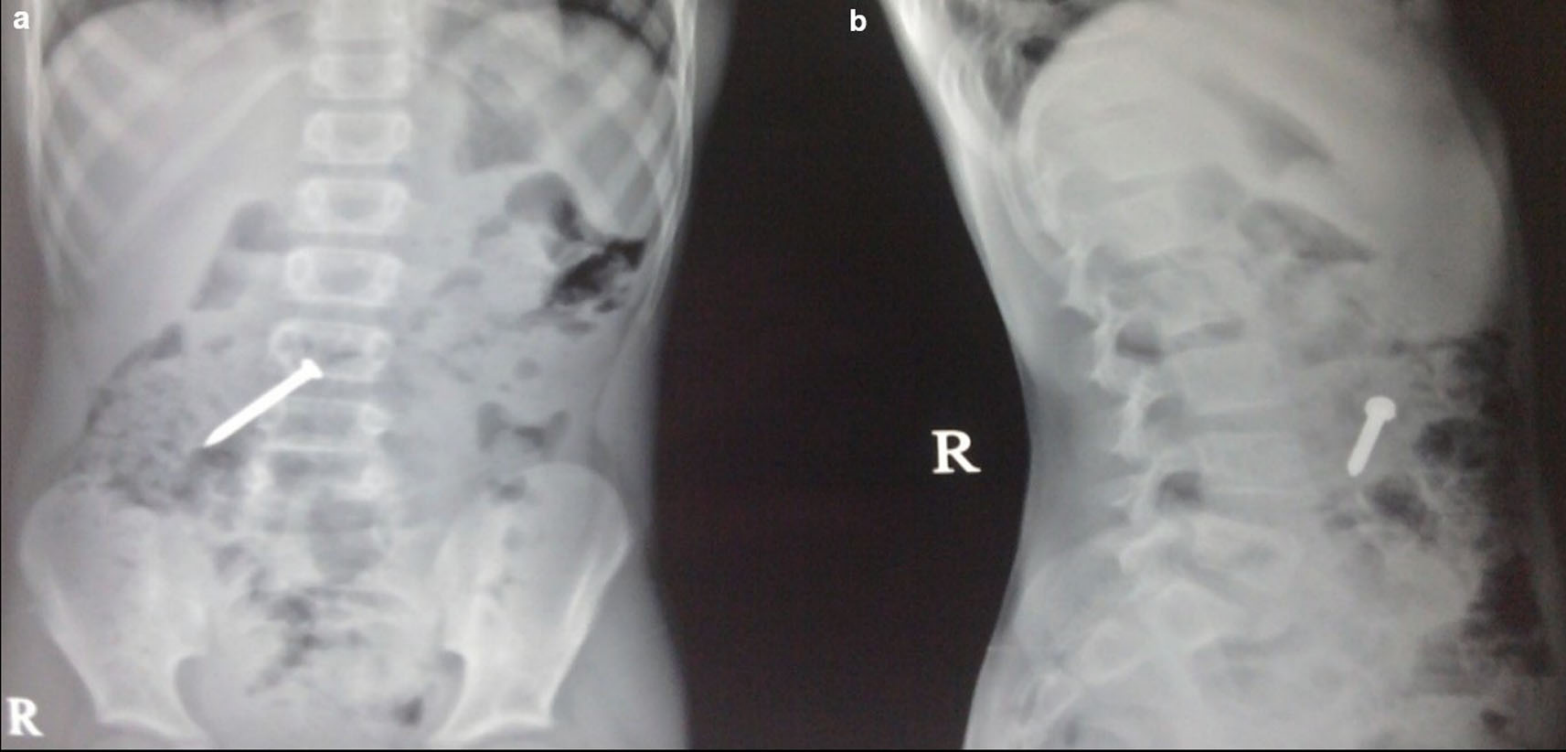
a & b: Radiograph of the abdomen in frontal and lateral views reveals a nail at the level of the L4 and L5 vertebrae in the gastrointestinal tract.

**Figure 3.**
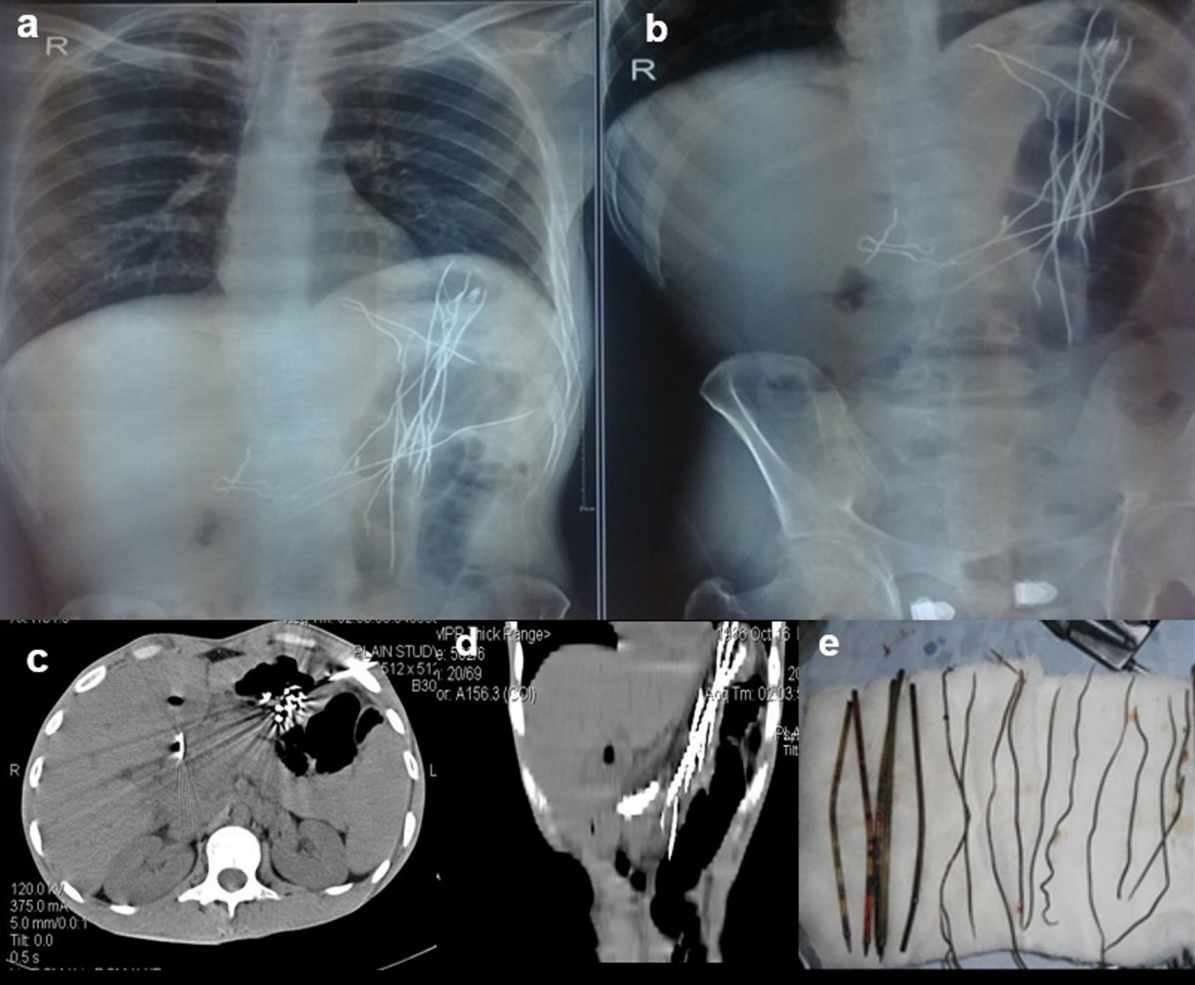
a-e: A Radiograph of the chest and abdomen reveal multiple linear radio-opacities in the left hypochondriac and lumbar quadrants of the abdomen (a & b), and a plain CT scan axial and sagittal image shows multiple hyperdense linear metallic foreign bodies within the gastric lumen, many piercing the gastric wall partially without any e/o perforation (c & d). **On laparoscopy, multiple refills of the pen and wires were found in the stomach, which were removed (e)**.

The swallowing of foreign bodies is a common condition in children and mentally challenged individuals. ^3^^–^^5^ Fortunately, most ingested objects move through the digestive system without causing any problems ( Figure 1a,b). Sharp and elongated objects can pass uneventfully ( Figure 2a,b); however, they can pierce the mucosal lining and seriously damage or completely perforate the intestinal wall ( Figure 3a-e). The object may just partially puncture the gut wall, resulting in a chronic inflammatory condition with few symptoms that is diagnosed months or years later. ^5^^–^^7^

**Figure 4.**
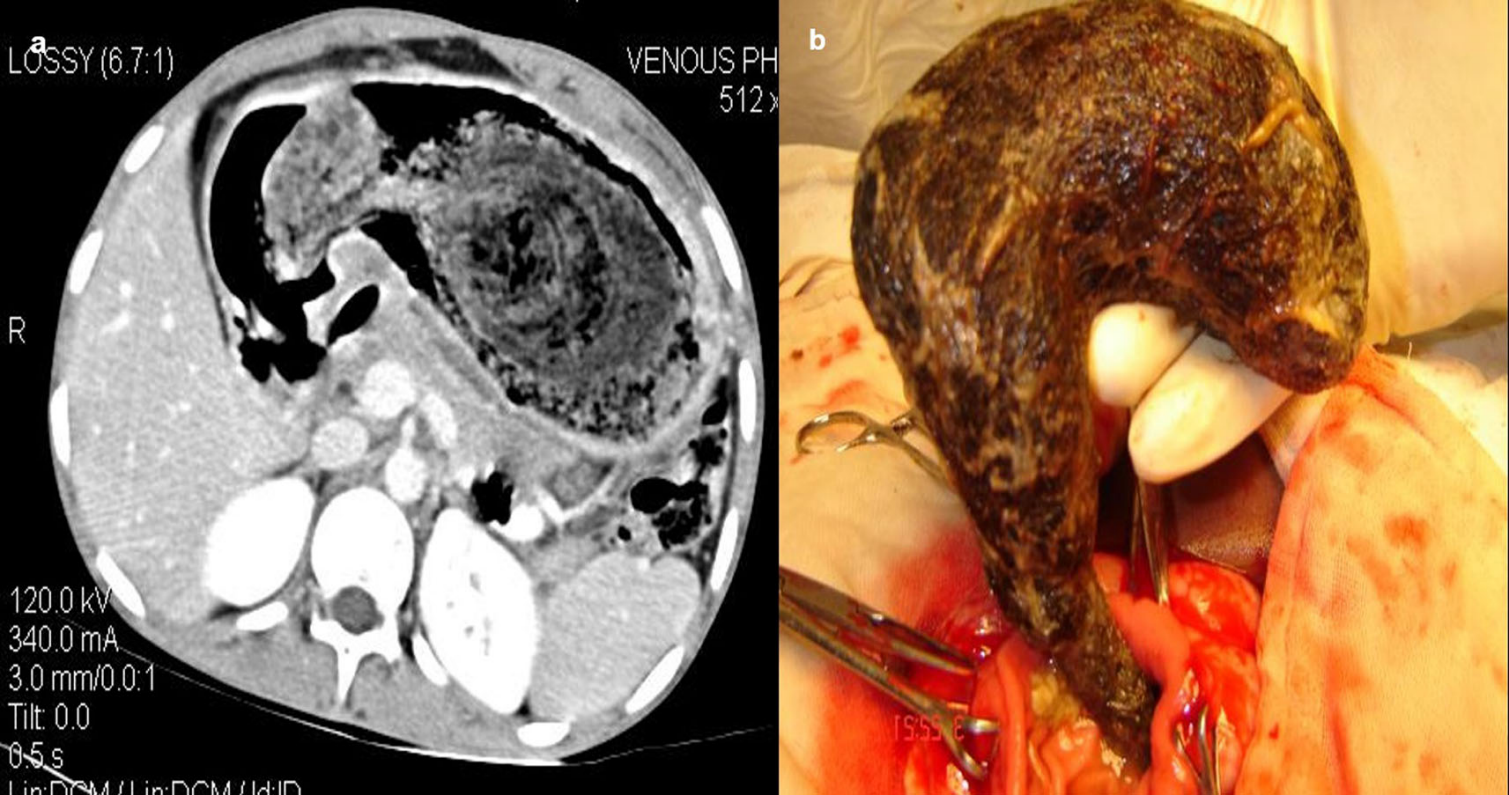
a & b: Contrast enhanced Computed tomography (CECT) axial section reveals a heterogeneous lamellated non-enhancing soft tissue density mass intraluminally in the stomach, conforming to its shape and extending into the antrum and pylorus -trichobezoar (a). Gastrotomy revealed the ball of hair in the stomach (b).

When a patient cannot provide a sufficient history or has swallowed things that are not naturally radio-opaque, the diagnosis of an ingested foreign body is frequently missed. If a foreign body is suspected and is not visible on a Radiograph because of its radiolucent nature, a CT scan of the abdomen or chest may be beneficial ^8^ ( Figure 4a,b).

Sometimes you may not have a proper history of the ingestion of sharp objects. When a patient has a history of ingesting a foreign body, whether it is an adult or a kid, they should be checked for the entire body, from the base of the skull to the anus, from the nasopharynx to the rectum. The hunt for other foreign bodies should not stop just because one has been discovered because youngsters are particularly prone to eating items in multiples. ^9^

### Foreign body insertions

**Figure 5.**
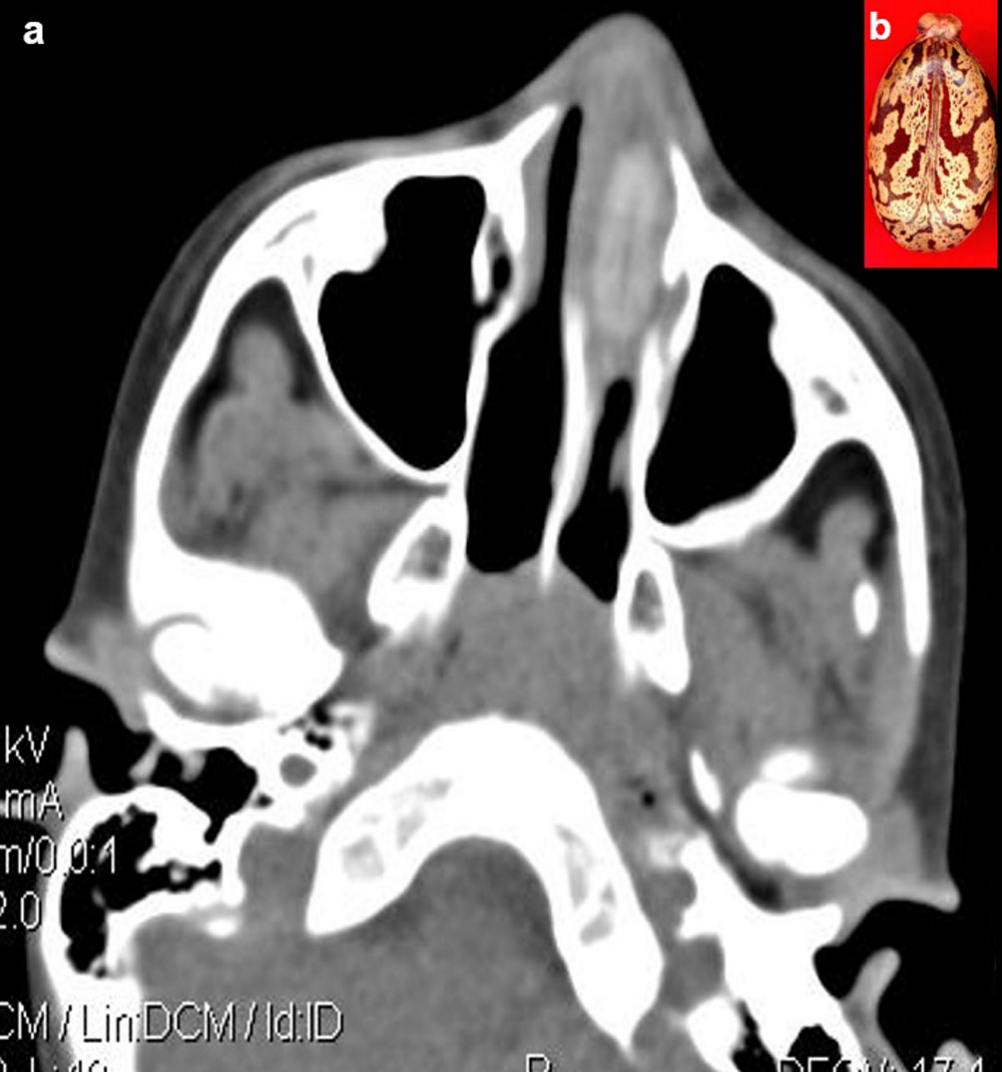
a & b: CT PNS axial section revealed a non-enhancing hyperdense foreign body in the left nasal cavity (a) Castor seed (b).

**Figure 6.**
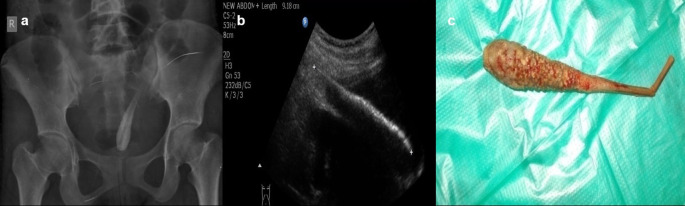
a–c: Radiograph Pelvis A-P view revealed long radio-opacity in the bladder with a radiolucent center - foreign body (a). USG revealed a linear hyperechoic foreign body that penetrated the anterior wall of the bladder (b). The removed foreign body was a long wooden stick with cotton wrapped around it (c).

The rectum, vagina, urethra, ear, and nose are common places for foreign items to be inserted. These are especially common in children ( Figure 5a,b) but can also be seen in adults. The deposition of mineral salts is especially likely to occur in foreign bladder substances, resulting in the formation of bladder calculi ( Figure 6a,c). In fact, when a child or young adult develops a bladder calculus, the presence of an embedded foreign body should be suspected. ^10^

### Foreign body injuries

Most people may have experienced at least one or two minor injury incidents, such as falls, abrasions, cuts, scrapes, and burns. Few of them may have experienced injuries from firearms and may have experienced puncture wounds from splinters, thorns, needles, or glass. ^2^

**Figure 7.**
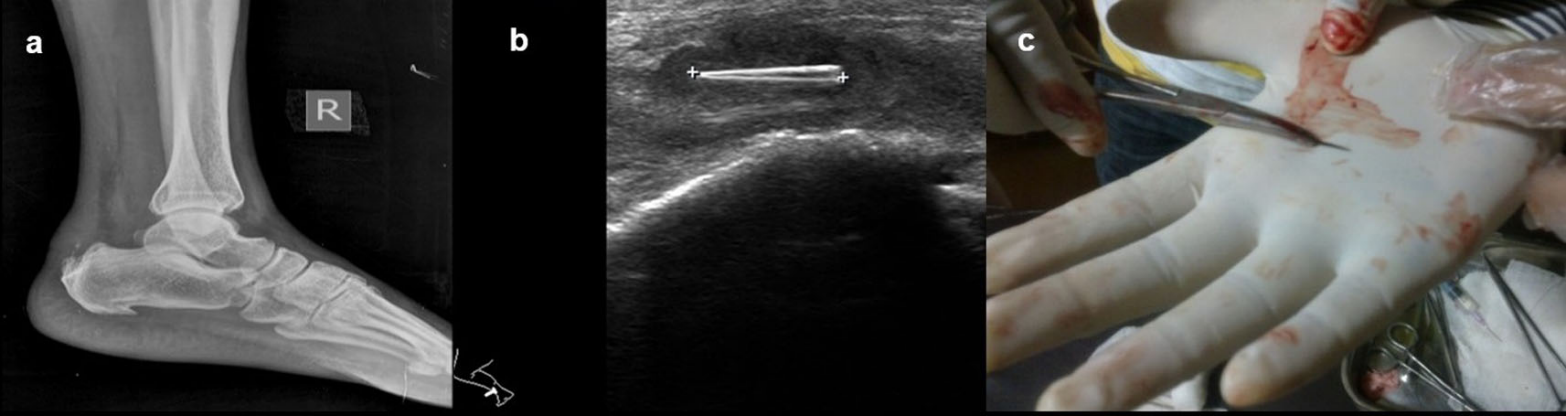
a–c: A radiograph of the lateral view of the right foot revealed calcific tendinitis of the Achilles tendon with thickening of the Kager fat pad and fat stranding (a). USG revealed that a well-defined thorn visualized in the Achilles tendon with associated changes of tendinitis (b). Thorn removed under ultrasound guidance (c).

On ultrasound, all foreign bodies in soft tissue are initially hyperechoic. Sonography is important for the correct localization of all kinds of soft tissue foreign bodies and the detection of non-radiopaque foreign bodies. Accurate localization can help minimize surgical exploration and can also direct the percutaneous removal of a foreign body ^11^ ( Figure 7a,b)

**Figure 8.**
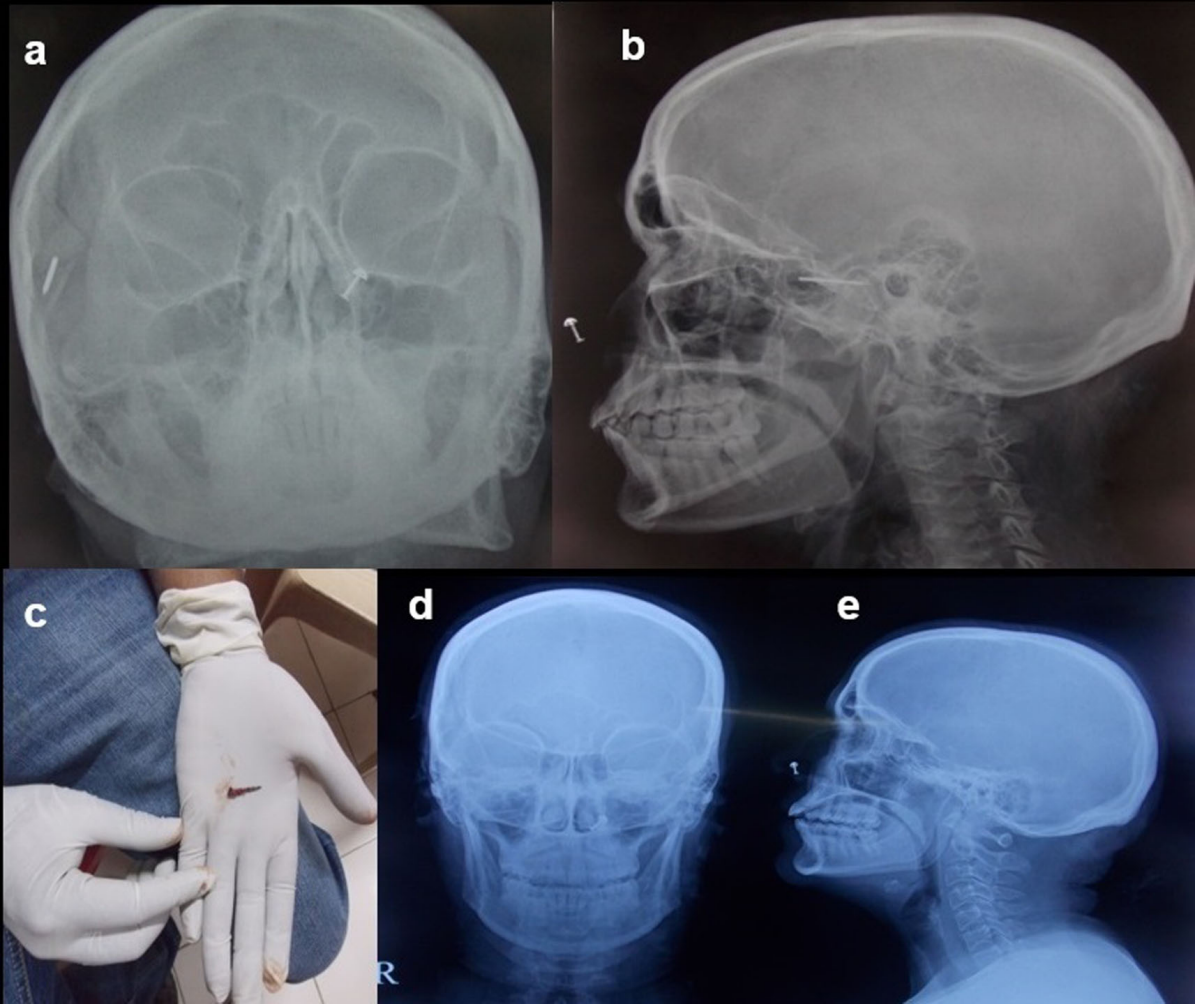
a-e: Radiograph skull A-P & Lateral view showed a radiodense nail-like structure on right side of scalp (a,b). **The nail removed under ultrasound guidance (c). Normal radiograph skull A-P & Lateral view post removal of the foreign body (d,e)**.

Some metallic foreign bodies can be accidentally diagnosed during an MRI or CT study due to artefacts or sometimes due to pain as they enter the magnetic field ^12^ ( Figure 8a,b).

**Figure 9.**
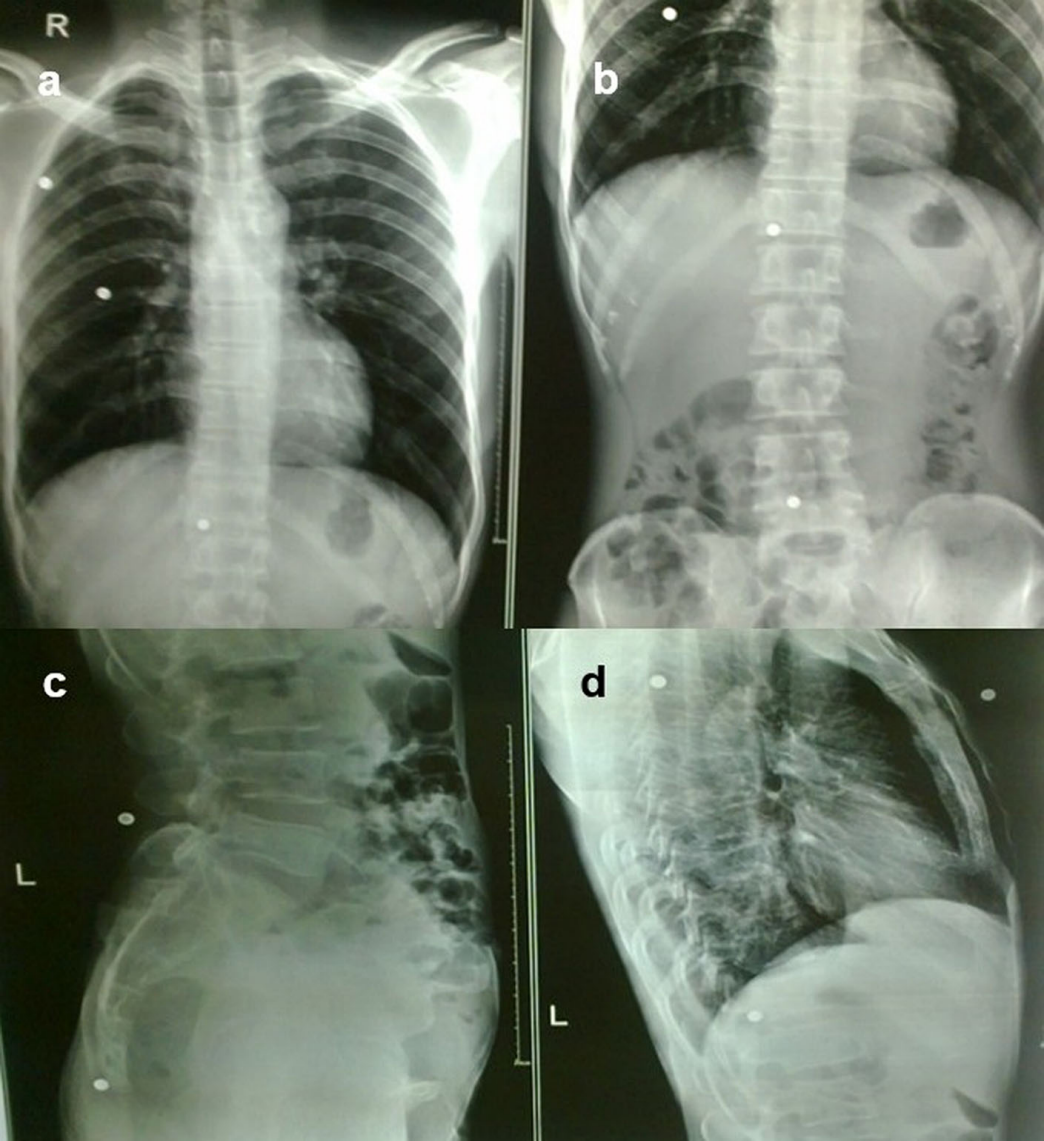
a–d: Radiograph of the chest and abdomen in frontal and lateral views revealed multiple radiodense pellets in the subcutaneous soft tissue of the thorax and abdomen.

The gauge of a shotgun pellet determines its size, the higher the number, the smaller the pellet. Serious soft tissue and bone damage can result from the combined mass striking a target close to the gun barrel ( Figure 9a-d). Because steel pellets are ferromagnetic, they could move dangerously if such a patient with embedded steel pellets was exposed to a magnetic field, making magnetic resonance imaging potentially dangerous. ^2^

**Figure 10.**
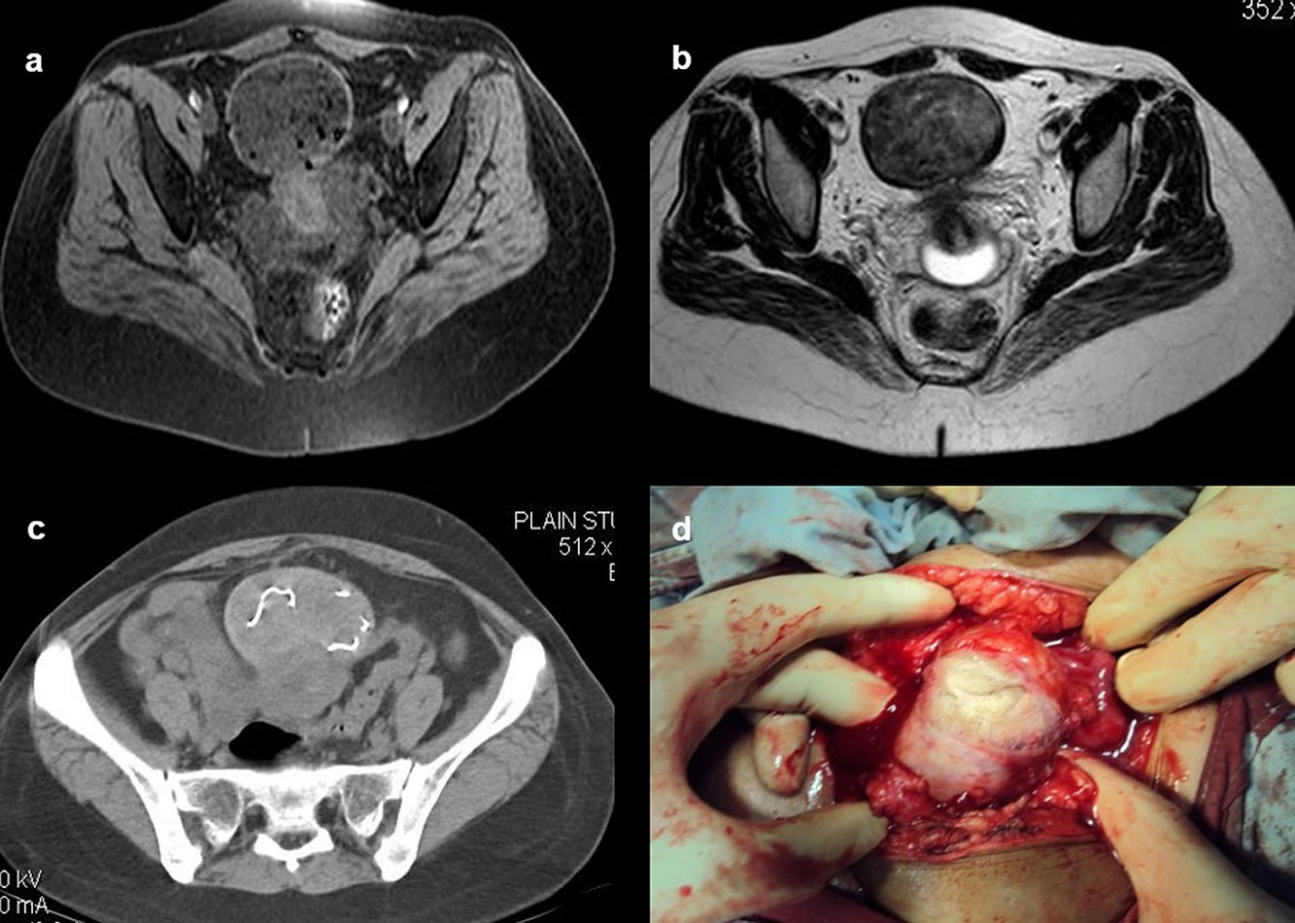
a-d: MRI axial T2 and T1 Fat Sat images shows heterogeneous altered signal intensity soft tissue mass anterior to the uterus with multiple hypointense foci within (a, b) CT showed multiple linear metallic strings within a soft tissue density mass anterior to the uterus-gossypiboma (c). The surgical sponge was removed at surgery (d).

After surgery, not infrequently, patients have surgical items inside their bodies. Surgical drains, wound gauze packs, bandages, skin staples, small surgical staples, intra-arterial, intravenous, intra-spinal, and intraabdominal catheters are among the postoperative supplies that are most frequently seen. Other uncommon materials, such as retained abdominal sponges ( Figure 10a-d) and needles, that were unintentionally left behind after surgery, are challenging to find clinically and radiographically because patients have vague symptoms, these objects are difficult to see on radiographs, and the radiologist and referring physician have a low level of suspicion for such objects. The nursing staff may perform a comprehensive sponge count at the conclusion of a surgical procedure and identify any remaining surgical sponges right away. A misplaced sponge may not be identified for months or even years after surgery if it is not found at that time. The foreign body reaction to a surgical sponge left inside the body for a long time is frequently called a gossypiboma. The sponge’s cotton matrix is what creates the foreign body reaction’s nidus. There is the development of a foreign body granuloma with surrounding fibrosis and retraction around the cotton nidus. Many people have no symptoms, and the retained sponge is often only unintentionally found when the patient has a radiological examination for another reason. ^13^^–^^17^

## Conclusion

Foreign bodies are interesting, and most of them are diagnosed incidentally in various parts of the human body and can cause significant harm if not properly managed. The diagnosis and management of foreign bodies can be challenging and require a high index of suspicion. Imaging studies such as Radiographs, CT scans, USG, and magnetic resonance imaging can be helpful in detecting and localizing foreign bodies. The management of foreign bodies can involve a variety of interventions, including endoscopy, surgical exploration, and percutaneous removal. Prevention is also key, particularly in children and mentally handicapped adults who are at increased risk of foreign body ingestion or insertion. It is important for healthcare providers to be aware of the potential for foreign bodies and to maintain a high level of vigilance when evaluating patients. Ultimately, early detection and appropriate management can prevent serious complications and improve patient outcomes.

## Data Availability

All data underlying the results are available as part of the article and no additional source data are required. CARE guidelines for case reports: 13-item checklist
