# Supplementary material for: Few incidentally found interesting foreign objects in human body: a case series
Source: F1000Res. 2024 Jul 18;12:1306. Originally published 2023 Oct 11. [Version 3] doi: 10.12688/f1000research.134773.3 (PMC11927391; doi:10.12688/f1000research.134773.3)
Supplement: Peer review history

# Reviewer report: Reviewer response for version 3

**DOI:** 10.5256/f1000research.168320.r371635

**Published:** 2025-03-20

**Authors:** Hüseyin Fatih Sezer

**Affiliations:** 1 Department of Thoracic Surgery, Kocaeli University Faculty of Medicine, Kocaeli, Turkey

I read a nice article about foreign bodies in different parts of the body. I liked your treatment suggestions as well as case examples and diagnostic methods. I think it will be of interest to readers. I would like to read a more expanded version of your series with new cases in the future.

Are enough details provided of any physical examination and diagnostic tests, treatment given and outcomes?

Yes

Is the background of the cases’ history and progression described in sufficient detail?

Yes

Is sufficient discussion included of the importance of the findings and their relevance to future understanding of disease processes, diagnosis or treatment?

Yes

Is the conclusion balanced and justified on the basis of the findings?

Yes

Reviewer Expertise:

thoracic surgery, tracheobronchial foreign body

I confirm that I have read this submission and believe that I have an appropriate level of expertise to confirm that it is of an acceptable scientific standard.

# Response

**Published:** 2025-03-20

**Authors:** ANAND HATGAONKAR

**Affiliations:** Radiodiagnosis, Datta Meghe Medical College, Nagpur, NAGPUR, MAHARASHTRA, India

Thanks for all the appreciation and review of this decade long work about interesting foreign bodies.

# Reviewer report: Reviewer response for version 3

**DOI:** 10.5256/f1000research.168320.r371631

**Published:** 2025-03-20

**Authors:** Karthik Krishna Ramakrishnan

**Affiliations:** 1 Saveetha Medical College and Hospital, Saveetha Institute of Medical and Technical Sciences (SIMATS), Saveetha University, Chennai, India

This case series presents ten interesting cases of foreign body ingestion, insertion, and injury. The authors effectively illustrate the diagnostic role of different imaging modalities such as X-rays, CT, MRI, and ultrasound. The study spans a wide range of foreign bodies, from swallowed coins and screws to retained surgical sponges and penetrating injuries. The discussion covers diagnostic challenges, complications, and management approaches, making the study informative for radiologists and clinicians.

**Recommendations**

**1. Improve Clinical Examination Details**

- Some cases lack thorough documentation of physical findings, particularly negative findings. A more structured clinical examination section for each case would strengthen the manuscript.

**2. Expand on Management Strategies**

- Although the authors have included treatment details, they should incorporate more references to established guidelines such as the **European Society of Gastrointestinal Endoscopy (ESGE) guidelines** on foreign body removal.
- The discussion should clarify decision-making criteria for endoscopic versus surgical intervention.

**3. Update Language for Sensitivity**

- Certain terms such as "mentally handicapped" are outdated and should be replaced with **"people with intellectual disability"** to align with modern medical terminology and ethical standards.

**4. Strengthen the Discussion on Risk Factors**

- The article would benefit from a discussion on common risk factors for foreign body ingestion, such as psychiatric conditions, neurological impairments, and substance use disorders.

**5. Balance the Conclusion**

- The conclusion should not only summarize findings but also suggest potential areas for further research, particularly regarding early diagnosis and prevention strategies.

**Required Changes for Scientific Soundness**

 To ensure the article meets the highest scientific standards, the following points must be addressed:

 ✔ Add missing clinical examination details for some cases.

 ✔ Include references to **established guidelines** on endoscopic and surgical management.

 ✔ Revise outdated terminology to align with modern medical ethics.

 ✔ Expand the discussion on **risk factors** for foreign body ingestion and insertion.

 ✔ Strengthen the **conclusion** with key takeaways and research gaps.

 Overall, this is an interesting and well-illustrated case series that will benefit from additional refinement and evidence-based referencing to enhance its scientific value.

Are enough details provided of any physical examination and diagnostic tests, treatment given and outcomes?

Partly

Is the background of the cases’ history and progression described in sufficient detail?

Yes

Is sufficient discussion included of the importance of the findings and their relevance to future understanding of disease processes, diagnosis or treatment?

Partly

Is the conclusion balanced and justified on the basis of the findings?

Partly

Reviewer Expertise:

Radiology, Neuroradiology, Oncologic Imaging, Emergency Radiology, Cross-sectional Imaging, Musculoskeletal Imaging, Soft Tissue Tumors, CT Imaging, Doppler Ultrasound.

I confirm that I have read this submission and believe that I have an appropriate level of expertise to confirm that it is of an acceptable scientific standard, however I have significant reservations, as outlined above.

# Reviewer report: Reviewer response for version 3

**DOI:** 10.5256/f1000research.168320.r304477

**Published:** 2024-08-02

**Authors:** Giacomo Calini

**Affiliations:** 1 Department of Surgery of the Alimentary Tract, Universitaria di Bologna, Bologna, Italy

I acknowledge the authors for the addition of a paragraph explaining the current treatment indicated for foreign bodies. However, the citations are inappropriate. For example, you used a single StatPearl about "Pediatric Foreign Body Ingestion" as a reference when speaking of all-ages foreign body ingestion while failing to add (even if indicated in the previous peer review) major guidelines and most recent scientific data available for endoscopic management of foreign body. This needs to be amended.

 In addition, you failed to modify the language as previously indicated. Some terms, like mentally handicapped, are outdated in medicine because they carry an offensive intent. Revise these terms with more appropriate terminology. eg, people with intellectual disability.

 This is mandatory considering the F1000 policy stating: "Appropriate non-stigmatizing language should be used"  https://f1000research.com/about/policies#ethpol

 The response to the previous revision did not explain the absence of these modifications required. Please revise the manuscript properly or rebut it.

Are enough details provided of any physical examination and diagnostic tests, treatment given and outcomes?

Yes

Is the background of the cases’ history and progression described in sufficient detail?

Yes

Is sufficient discussion included of the importance of the findings and their relevance to future understanding of disease processes, diagnosis or treatment?

Partly

Is the conclusion balanced and justified on the basis of the findings?

Partly

Reviewer Expertise:

surgery, endoscopy,

I confirm that I have read this submission and believe that I have an appropriate level of expertise to confirm that it is of an acceptable scientific standard, however I have significant reservations, as outlined above.

## References

[1] : Removal of foreign bodies in the upper gastrointestinal tract in adults: European Society of Gastrointestinal Endoscopy (ESGE) Clinical Guideline. *Endoscopy* .2016;48(5) : 10.1055/s-0042-100456 489-96 10.1055/s-0042-100456 26862844

[2] : Foreign body and caustic ingestions in children: A clinical practice guideline. *Dig Liver Dis* .2020;52(11) : 10.1016/j.dld.2020.07.016 1266-1281 10.1016/j.dld.2020.07.016 32782094

[3] : Endoscopic failure for foreign body ingestion and food bolus impaction in the upper gastrointestinal tract: an updated analysis in a European tertiary care hospital. *Eur J Gastroenterol Hepatol* .2023;35(9) : 10.1097/MEG.0000000000002602 962-967 10.1097/MEG.0000000000002602 37395211

# Reviewer report: Reviewer response for version 2

**DOI:** 10.5256/f1000research.166940.r287317

**Published:** 2024-06-28

**Authors:** Kai Sheng Hsieh

**Affiliations:** 1 Structural/Congenital Heart Disease and Ultrasound Center, Children's Hospital, China Medical University,, China Medical University--Children's Hospital, TaiChung, Taiwan, Taiwan

The authors have revised the manuscript according to my comments and questions. I will thus suggest acceptance of revised manuscript for indexing. Thanks.

Are enough details provided of any physical examination and diagnostic tests, treatment given and outcomes?

Partly

Is the background of the cases’ history and progression described in sufficient detail?

Partly

Is sufficient discussion included of the importance of the findings and their relevance to future understanding of disease processes, diagnosis or treatment?

Partly

Is the conclusion balanced and justified on the basis of the findings?

Partly

Reviewer Expertise:

Pediatrics, Pediatric Cardiology, Pediatric Emergency-Critical Care, Pediatric Pulmonology, Neonatology, Perinatology, Ultrasound/Echocardiography

I confirm that I have read this submission and believe that I have an appropriate level of expertise to confirm that it is of an acceptable scientific standard.

# Reviewer report: Reviewer response for version 2

**DOI:** 10.5256/f1000research.166940.r287318

**Published:** 2024-06-10

**Authors:** Giacomo Calini

**Affiliations:** 1 Department of Surgery of the Alimentary Tract, Universitaria di Bologna, Bologna, Italy

The authors addressed none of the comments indicated in my previous peer review. No explanation about it was provided in the amendments from the authors

Are enough details provided of any physical examination and diagnostic tests, treatment given and outcomes?

Yes

Is the background of the cases’ history and progression described in sufficient detail?

Yes

Is sufficient discussion included of the importance of the findings and their relevance to future understanding of disease processes, diagnosis or treatment?

Partly

Is the conclusion balanced and justified on the basis of the findings?

Partly

Reviewer Expertise:

surgery, endoscopy,

I confirm that I have read this submission and believe that I have an appropriate level of expertise to confirm that it is of an acceptable scientific standard, however I have significant reservations, as outlined above.

# Response

**Published:** 2024-06-23

**Authors:** ANAND HATGAONKAR

**Affiliations:** Radiodiagnosis, Datta Meghe Medical College, Nagpur, NAGPUR, MAHARASHTRA, India

Dear Giacomo Calini, 

 Thanks for the review and suggestion. I am prepairing next version with the amendments in the article with inclusion of treatment part. The changes are yet to be uploaded. 

 Thanks and regards,

 Dr Anand Hatgaonkar

# Reviewer report: Reviewer response for version 1

**DOI:** 10.5256/f1000research.147853.r278383

**Published:** 2024-05-30

**Authors:** Giacomo Calini

**Affiliations:** 1 Department of Surgery of the Alimentary Tract, Universitaria di Bologna, Bologna, Italy

I praise the authors for this interesting and broad case series and review of novel and rare episodes of foreign objects. 

 In the case of high-risk ingested foreign bodies endoscopy is required. Therefore, my advice is to discuss this treatment option, including the publications below (Ref 1-3).

 Also, terms like mentally handicapped, are outdated in medicine as they generally carry an offensive intent. Revise these term with a more appropriate terminology. eg, people with intellectual disability

Are enough details provided of any physical examination and diagnostic tests, treatment given and outcomes?

Yes

Is the background of the cases’ history and progression described in sufficient detail?

Yes

Is sufficient discussion included of the importance of the findings and their relevance to future understanding of disease processes, diagnosis or treatment?

Partly

Is the conclusion balanced and justified on the basis of the findings?

Partly

Reviewer Expertise:

surgery, endoscopy,

I confirm that I have read this submission and believe that I have an appropriate level of expertise to confirm that it is of an acceptable scientific standard, however I have significant reservations, as outlined above.

## References

[1] : Removal of foreign bodies in the upper gastrointestinal tract in adults: European Society of Gastrointestinal Endoscopy (ESGE) Clinical Guideline. *Endoscopy* .2016;48(5) : 10.1055/s-0042-100456 489-96 10.1055/s-0042-100456 26862844

[2] : Foreign body and caustic ingestions in children: A clinical practice guideline. *Dig Liver Dis* .2020;52(11) : 10.1016/j.dld.2020.07.016 1266-1281 10.1016/j.dld.2020.07.016 32782094

[3] : Endoscopic failure for foreign body ingestion and food bolus impaction in the upper gastrointestinal tract: an updated analysis in a European tertiary care hospital. *Eur J Gastroenterol Hepatol* .2023;35(9) : 10.1097/MEG.0000000000002602 962-967 10.1097/MEG.0000000000002602 37395211

# Response

**Published:** 2024-06-24

**Authors:** ANAND HATGAONKAR

**Affiliations:** Radiodiagnosis, Datta Meghe Medical College, Nagpur, NAGPUR, MAHARASHTRA, India

Thanks for the review, 

 As per your suggestion, we have updated information about the treatment strategies used to remove different foreign objects from human body.

# Reviewer report: Reviewer response for version 1

**DOI:** 10.5256/f1000research.147853.r237799

**Published:** 2024-05-20

**Authors:** Kai Sheng Hsieh

**Affiliations:** 1 Structural/Congenital Heart Disease and Ultrasound Center, Children's Hospital, China Medical University,, China Medical University--Children's Hospital, TaiChung, Taiwan, Taiwan

This article entitled "Few incidentally found interesting foreign objects in human body: a case series" was reviewed. The article summarized 10 cases of different forms of foreign body retention within the human body . Their ages ranged from 4 years old to 50 years old. Multiple modalities of diagnostic equipment were employed for imaging purpose. With these remarkable experience, the authors conclude that physicians should be alerted about the possibility of foreign body retention within the human body. Although the article is well written, I have the following comments for authors to respond:

 1. Please add the duration of symptom(s) for each individual case. Most of the 10 cases lack this data (the information is present only in cases 3,4,7 and 10) .

 2. Please add the concise essential findings of physical examination (significant positive nd negative findings).

 3. Please add some sentences about the choices of diagnostic imaging ( Plain X-rays, CT , MRI and ultrasound) in the Discussion section.

 4. Please add some sentences regarding the analysis of “risk factors” including those present in this article and those mentioned in other literatures in the Discussion section.

Are enough details provided of any physical examination and diagnostic tests, treatment given and outcomes?

Partly

Is the background of the cases’ history and progression described in sufficient detail?

Partly

Is sufficient discussion included of the importance of the findings and their relevance to future understanding of disease processes, diagnosis or treatment?

Partly

Is the conclusion balanced and justified on the basis of the findings?

Partly

Reviewer Expertise:

Pediatrics, Pediatric Cardiology, Pediatric Emergency-Critical Care, Pediatric Pulmonology, Neonatology, Perinatology, Ultrasound/Echocardiography

I confirm that I have read this submission and believe that I have an appropriate level of expertise to confirm that it is of an acceptable scientific standard, however I have significant reservations, as outlined above.

# Response

**Published:** 2024-06-23

**Authors:** ANAND HATGAONKAR

**Affiliations:** Radiodiagnosis, Datta Meghe Medical College, Nagpur, NAGPUR, MAHARASHTRA, India

Dear Kai Sheng Hsieh,

 Thanks for the review,

 I am really thankful to you for nice and thoughtful review. Your review had helped me in making this article more comprehensive. As it is all about imaging I have added all necessary details about it.

 Thanks & Regards,

 Dr. Anand Hatgaonkar
